# Oligometastatic disease and visceral resections in advanced malignant melanoma: a propensity-matched analysis

**DOI:** 10.1007/s00423-023-02804-9

**Published:** 2023-01-21

**Authors:** Alexander Urbanski, Julia Minnemann, Cornelia Mauch, Thomas Schmidt, Nicole Kreuzberg, Max Schlaak, Christiane J. Bruns, Dirk L. Stippel, Roger Wahba

**Affiliations:** 1 Department of General, Visceral, Cancer and Transplant Surgery, Faculty of Medicine and University Hospital of Cologne, Cologne, Germany; 2 Department of Dermatology and Venereology, Faculty of Medicine and University Hospital of Cologne, Cologne, Germany; 3 Department of Dermatology, Venereology and Allergology, Charité – Universitätsmedizin Berlincorporate member of Freie Universität Berlin and Humboldt-Universität Zu Berlin, Berlin, Germany

**Keywords:** Melanoma, Malignant melanoma, Oligometastasis, Visceral resection, Visceral metastasis, Surgery

## Abstract

**Purpose:**

Malignant melanoma is among the tumours with the highest increase in incidence of solid tumours in Germany. While most patients are diagnosed at an early stage and show a good prognosis, advanced stages of malignant melanoma are accompanied with a poor prognosis and limited treatment options. Comparable to other tumour entities, the resection of visceral metastases could lead to a better prognosis. Supplementary, the subgroup of oligometastatic patients might benefit from surgical therapy to a greater extent.

**Methods:**

This retrospective study analysed 351 patients treated between 2006 and 2017 at the University Hospital of Cologne. A total of 121 patients showed visceral metastases, with which we compared patients with a diffuse tumour spread to patients in an oligometastatic state. Furthermore, we evaluated the effect of visceral resection of oligometastatic, malignant melanoma.

**Results:**

Our analysis showed that patients with an oligometastatic malignant melanoma had a significantly better prognosis than patients with a diffuse pattern of metastases, if they showed visceral metastases. Furthermore, the resection of visceral metastases leads to a significant gain in median overall survival time (13.6 vs. 34.2 months) and in progression-free survival (9.6 vs. 3.8 months).

**Conclusion:**

The resection of visceral metastases is a rational treatment option in advanced malignant melanoma. Although our study is limited by a small cohort of patients (*n* = 18), we believe that the resection of visceral metastases will be fundamental in the treatment of malignant melanoma. In particular, patients in an oligometastatic stage could be an eligible group for surgical treatment.

**Supplementary Information:**

The online version contains supplementary material available at 10.1007/s00423-023-02804-9.

## Introduction

Malignant melanoma (MM) is rising in incidence in the western world [1, 2] and is estimated to account for approximately 1% of cancer-related deaths in the USA in 2019 [2]. At an early stage, treatment consists of complete excision of the melanoma with consecutive follow-up of the patient [3]. Early detected MM (TIa/TIb stage) has a 10-year survival rate close to 100% [4, 5]. The detection of only one metastatic lymphnode (LN) significantly lowers to 88% for 10-year survival [5]. When comparing early stages to stage IV MM, which is defined by any distant metastasis, the prognosis is even more drastically reduced. Within this group, patients with visceral metastases (M1c) have the lowest survival rates. The current German S3 guidelines recommend a surgical resection of distant metastases whenever possible and when this does not lead to functional deficits [6]. Systemic treatment of malignant melanoma depends on potential mutations, such as BRAF, NRAS, and c-kit mutations, responsive for current therapeutics. For BRAF-positive patients (up to 50% of malignant melanoma), effective treatment options are now available [7]. The combination of a BRAF-inhibitor as dabrafenib with a MEK-inhibitor (such as trametinib) or a checkpoint-inhibtion therapy (such as pembrolizumab) can lead to a 1-year survival of up 72% and a median survival of up to 25 months [8, 9]. In patients without a targetable mutation, the performance of an immunotherapy (such as ipilimumab), favorably in combination with PD-1-antibody, can still lead to a median survival of almost 12 months [10]. In summary, due to the continous development of the systemic and targeted therapies, including checkpoint inhibitors, multikinase inhibitors and even oncologic virotherapy, survival of more than 5 years for patients with advanced malignant melanoma [11, 12] could be achieved.

However, the limited survival, especially in young patients, is not satisfactory, and different treatment options are still needed. Chemotherapy alone might stabilise the disease for a certain amount of time, but studies have not yet shown a benefit in survival [13]. Radiation therapy is only applied in a palliative setting [14, 15].

To further distinguish patients with a small amount of metastases from more widely disseminated cancers, the term “oligometastatic” was introduced [16]. The parental idea is to find a subset of patients in metastatic cancer, which is still eligible for a wider array of therapeutical options. Oligometastatic disease could be defined by a limited number and location of the metastases, which are amenable to regional treatment [16]. The most prominent example using the idea of “oligometatastatic diesaes” is the treatment of patients with metastastic colorectal cancer. Even if systemic therapy can result in sufficient tumour control, the resection of colorectal metastases remains the only potential cure with superior survival [17]. As an example, the implementation of surgical resection of colorectal liver metastases leads to a median 5-year survival of 40% today, with individual patients living more than 10 years after resection [18]. Analogous to this progress, visceral resection in oligometastatic MM seems feasible. It has been already shown that the number of organ sites with metastases and the sum of the lesion diameter of the metastasis are predictive for overall survival (OS) as well as for the progression-free survival (PFS) [1, 19]. While the benefit of surgical resections in oligometastatic stage IV melanoma in cohorts of 64 patients could be shown, only very few patients undergoing visceral resections were included [1]. There is still only very limited reliable data adressing the issue of visceral resections in oligometastatic MM. Identifying patients eligible for surgery is especially challenging. Further, an oligometastatic stage might be a positive prognostic factor in MM. The aim of this study was to further evaluate the influence of a surgical approach towards the prognosis in treatment of advanced malignant melanoma with visceral metastases and the possible impact of anoligometastic disease in comparison to a disseminated stage.

## Materials and methods

### Patients

For this retrospective study, we identified patients diagnosed with an advanced (stage IV according to the AJCC classification of 2009) cutaneus malignant melanoma, seen at the Department for Dermatology of the University Hospital of Cologne between 2006 and 2017. We limited our analysis to patients diagnosed with primary cutaneus melanoma. Generally, demographic and disease-specific data was collected (including date of diagnosis of the MM, date of reaching stage IV, localisation of metastasis, LDH levels, S100 levels, PFS, BMI, and date of death). Therapy options included chemotherapy, targeted therapy, local therapy, and immunotherapy. In addition, the timing of therapy (perioperative systemic therapy prior or following surgery) was analysed. The treatment with a BRAF or MEK-inhibitor was reviewed. Period of follow-up was 60 months.

Patients with visceral metastases were further grouped into oligometastic and non-oligometastatic cohorts. Oligometastic disease was defined as < = 5 metastases, according to the most common upper treshold in literature. Patients with lung metastases were excluded. All patients with visceral metastases were subgrouped to “receiving visceral resection” and to treated “conservatively”. Postoperative complications were analysed according to the Dindo-Clavien classification [20]. In surgical patients, the R-status and the number of resected metastases were analysed.

### Statistical analysis

The primary endpoint was OS. Distributions of time-to-event were estimated by median (95% confidence interval) and by Kaplan-Meier curves. Overall and progression-free survival was defined as the start with systemic first-line therapy. Missing data was censored either at the last known point of survival or censored in follow-up after 60 months.

Distributions of quantitative variables were summarised by valid count, mean, standard deviation or median, range, contingent on the presence of skewness/outliers. Qualitative variables were summarised by absolute and relative (percent) frequencies.

Association between any two qualitative/categorical variables was evaluated by the chi-square test, differences in survival distributions by the log-rank test.

Multivariable Cox-regression (including sex, age, BMI, BRAF) was used to explain variation in the time-to-event. The proportional hazards assumption was tested by introducing interaction with log-time as a time-dependent covariate. As cut-off for the assumption testing, we chose a *p*-value > 0.1/10%.

*P*-values < 0.05 were considered to indicate statistical significance. Calculations were performed using SPSS Statistics software (IBM Corp., Armonk, NY, USA).

Propensity scores for resection of visceral metastases were calculated for each patient with visceral metastases based on age and sex using a multivariable logistic regression model according to the method presented by Kuss et al. [21] and used to match all patients who received an operation with an equal number of patients who did not receive surgical treatment. For matching, we used a caliper width equal to 0.2 of the standard deviation of the logit of the propensity score [21, 22]. We used SPSS Statistics software. Matched-pair survival was estimated using the cox proportional hazards regression model (Table 1)

**Table 1:** Characteristics of all patients vs. patients with visceral metastases vs. patients with oligometastasis

	Stage IV	Visceral metastasis	Oligmetastatic
Characteristics	No. (%)	No. (%)	No. (%)
Total	351	121	18
Age			
Median	64	62	55
Sex			
Male	207 (59)	73 (60.3)	10 (55.6)
Female	144 (41)	48 (39.7)	8 (44.4)
BMI			
Median	26.04	25.67	25.81
BRAF			
Yes	157 (44.7)	58 (47.9)	10 (55.6)
No	152 (43.3)	47 (38.8)	8 (44.4)
No data	42 (12)	16 (13.2)	0
Number of metastatic sites *			
1	89 (25.4)	16 (13.2)	3 (16.7)
2	145 (41.3)	36 (29.4)	11 (61.1)
3	83 (23.6)	44 (36.1)	3 (16.7)
4	29 (8.3)	21 (17.2)	1 (5.6)
5	5 (1.4)	4 (3.3)	0
Visceral resection			
Yes	43	18	13
No	308	103	5

* Initial diagnosis stage IV

## Results

### Demographics

**Fig. 1:**
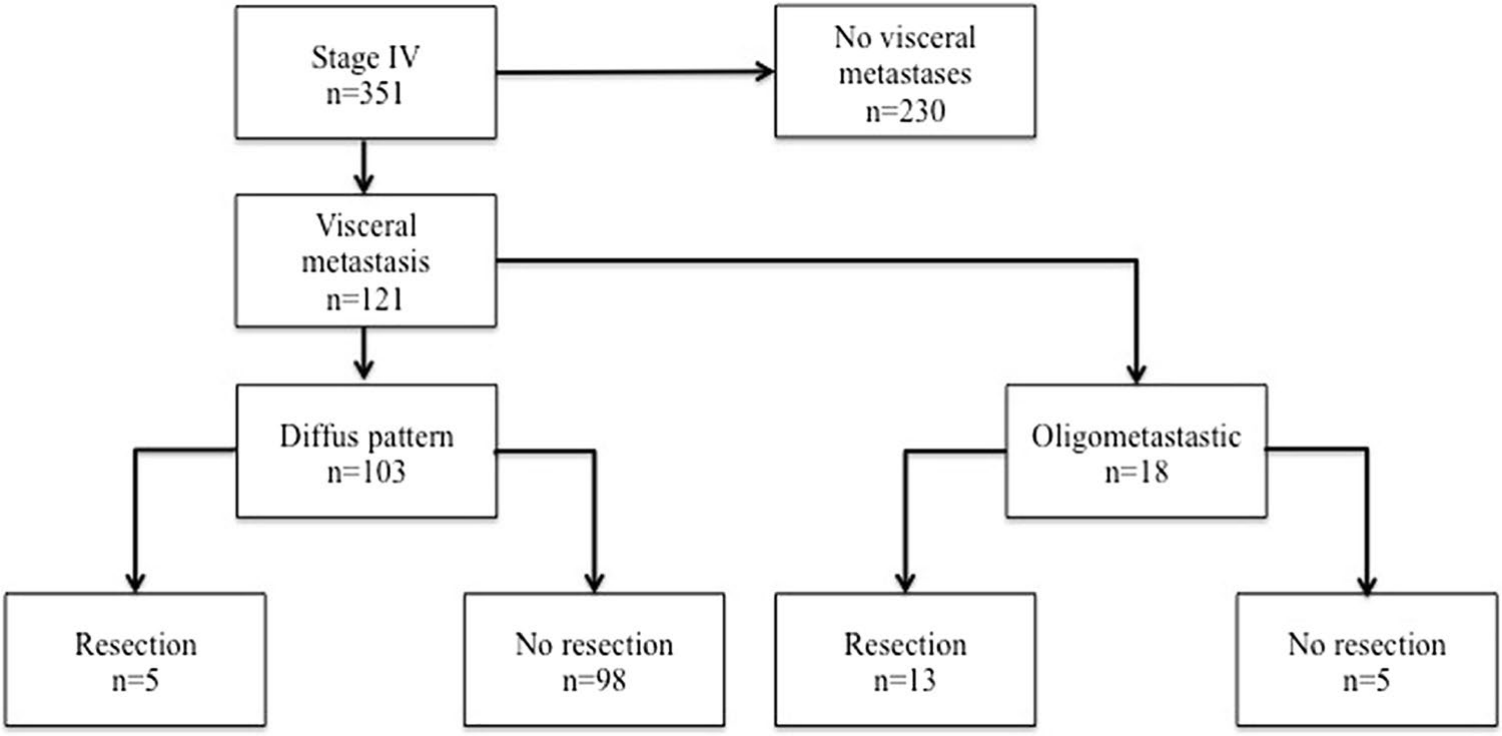
Patient selection and subgroups

Data of 351 patients with stage IV cutaneus maligant melanoma was analysed (Fig. 1). A total of 121 (34.5%) patients showed visceral metastases at initial diagnosis. The localisation of the visceral metastasis can be seen in Table 2. Patients’ demographics are shown in Table 1. In the group of patients with visceral metastases, 18 patients showed an oligometastatic disease, excluding patients with metastases to the lung (see Tables 1 and 2). Complete follow-up data was available for 73 patients (61%) for OS and for 107 (93.9%) of the patients for PFS.

**Table 2:** Location of the visceral metastases: all patients with visceral metastases vs. oligometastatic vs. diffuse vs. patients receiving visceral resection

	Visceral metastasis	Oligometastatic	Diffuse	Visceral resection	R0-resection
	No (%)	No (%)	No (%)	No (%)	No (%)
Total	121	18	103	18	13
Location visceral metastasis					
Liver	90 (74.3)	10 (56)	80 (77.6)	7 (39)	4 (31)
Adrenal gland	10 (8.3)	1 (6)	9 (8.7)	1 (6)	1 (8)
Stomach	4 (3.3)	1 (6)	3 (2.9)	1 (6)	1 (8)
Colon	4 (3.3)	1(6)	3 (2.9)	3 (17)	1 (8)
Small bowel	8 (6.6)	3 (17)	5 (4.9)	4 (22)	4 (31)
Spleen	12 (9.9)	1 (6)	11 (10.7)	1 (6)	1 (8)
Pancreas	5 (4.1)	2 (11)	3 (2.9)	1 (6)	1 (8)
Peritoneum	8 (6.6)	0 (0)	8 (7.8)	0 (0)	0

A BRAF-mutation was found in 58 (47.9%) patients, 47 (38.8%) patients did not show a BRAF-mutation, and for 16 (13.2%) patients, this information was not available.

### Treatment

Out of the 121 patients diagnosed with visceral metastases in stage IV melanoma, 18 (14.9%) patients received a resection of visceral metastases. A total of 13 (72%) patients of the surgical group showed an oligometastatic pattern of metastases compared to 5 patients (28%) with a disseminated pattern. Surgical resection was performed in median 4.5 weeks after diagnosis of stage IV malignant melanoma. A total of 14 patients were treated with resection of one metastasis, 1 patient with the resection of 2 metastases, and 1 patient with the resection of 3 metastases. In 2 patients, the exact number of resected metastases could not be retraced subsequently. The locations of the resected metastases are shown in Table 2.

Complications were graded according to the Dindo-Clavien classification. One patient, who underwent an atypical liver resection showed a grade IIIa complication that presented as an intraabdominal abscess. Another one patient showed a grade II complication (infection of the wound), following a resection of the bowel. No complications were reported for the remaining patients. In 13 (72%) patients, complete resection of metastases (R0 status) was achieved compared to an R1 status in 3 (17%) patients. In 2 (11%) patients, data concerning the R-status was missing.

Details about the modality and sequence of systemic therapy for all patients with visceral metastases are shown in Table 3.

**Table 3:** Patients with visceral metastases (*n* = 121); modality and sequence of systemic therapy

		Visceral resection	No visceral resection
	Preceding operation	Following operation	
	No (%)	No (%)	No (%)
Systemic therapy	7 (39)	10 (56)	91 (88.3)
No therapy	9 (50)	6 (33)	12 (11.7)
Signal transduction inhibitor	2 (11)	1 (6)	44 (42.7)
Immuno checkpoint inhibitor	2 (11)	4 (22)	53 (51.5)
Interferon	3 (17)	0	0
Chemotherapy	0	5 (28)	49 (47.6)
No data	2 (11)	2 (11)	2 (1.9)
Median OS	22.42 (95% CI 12.8–32.1)	23.4 (95% CI 13.0–33.7)	15.4 (95% CI 9.7–21.0)

### Survival

The overall median survival for patients in stage IV was 29.60 months (95% CI: 22.16–7.04) without visceral metastases and 16.76 (95% CI: 11.92–21.59) months for patients with visceral metastases (*p* = 0.001).

In patients with visceral metastases, a diffuse pattern of metastases led to a shorter OS (*p* = 0.003) (median OS, 13.60; 95% CI: 8.64–18.56) compared to an oligometastatic pattern (median OS was not reached in 60-month follow-up; 23.40 ± STE 7.77 75% quartile in oligometastatic disease vs. 4.07 ± 1.54 STE 75% quartile in diffuse disease).

**Fig. 2:**
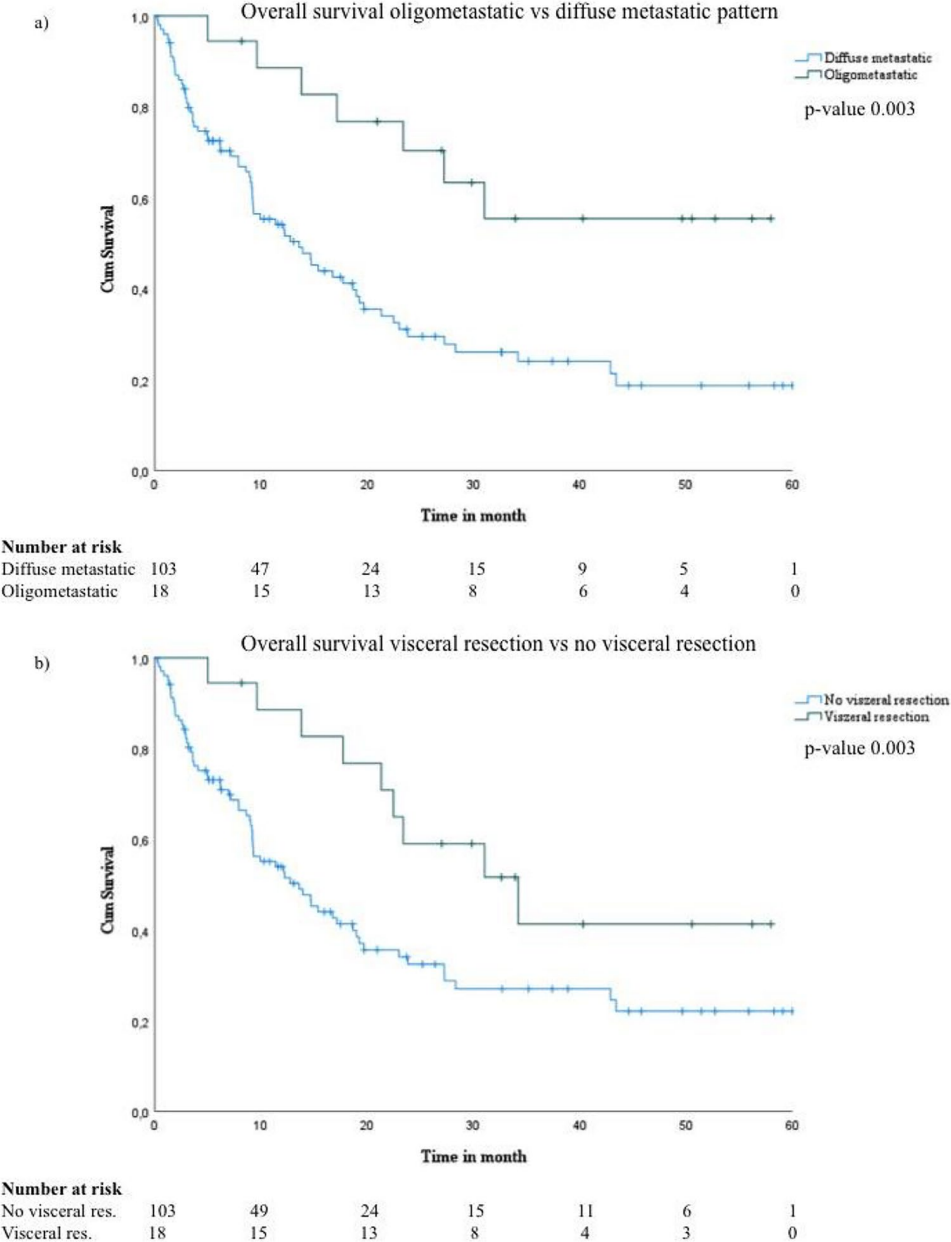
Overview of OS, **a** patients with stage IV malignant melanoma and visceral metastases with oligometastatic vs diffuse metastastic pattern and **b** these patients receiving visceral resection vs. no visceral resection

In patients with visceral metastases, the median OS was 13.60 (95% CI: 8.57–18.64) months without visceral resection and extended to 34.20 (95% CI: 17.91–50.50) months, if the patients received a visceral resection (*p* = 0.022) (Fig. 2).

Comparison between oligometastatic and diffuse metastatic patterns in the surgical group did not show a significant difference in median OS (29.83 (95% CI: 4.99–57.99) months in oligometastatic disease vs. 22.47 (95% CI: 2.0–24.9) months in diffuse metastatic disease (*p* = 0.249).

If a R0 situation could be achieved, the resection led to a significant benefit in median OS with 34.20 (95% CI: 4.99–57.99) months compared to 21.32 (95% CI: 9.28–33.36) month survival after R1/R2 resection (*p* = 0.04).

The analysis of the OS in the group receiving systemic therapy did not show any significant effects.

Matched pair analysis showed a significant impact (*p* = 0.016) on OS in the surgical group with a hazard ratio (HR) of 0.35, indicating a risk reduction of 65% by the treatment (95% CI: 0.149–0.825).

While BRAF mutation (*p* = 0.76), receiving BRAF (*p* = 0.14) or MEK inhibitors (*p* = 0.6) or BMI (*p* = 0.67) did not indicate a significant impact on OS in the matched pair analysis, patients with an oligometastatic pattern showed an significant (*p* = 0.025) advantage in OS with a hazard ratio (HR) of 0.34, indicating a risk reduction of 66% (95% CI: 0.13–0.87) (Table 4).

**Table 4:** Characteristics of matched pair analysis (MP): complete matched group (CM) vs. matched patients with oligometastatic (MP) vs. matched patients with visceral resection (MP) vs. matched patients with no visceral resection (MP)

	Visceral metastasis (CM)	Oligmetastatic (MP)	Visceral resection (MP)	No visceral resection (MP)
Characteristics	No. (%)	No. (%)	No. (%)	No. (%)
Total	36	14	18	18
Age				
Median	52	55	52.5	50
Sex				
Male	21 (58)	7 (50)	10 (56)	11 (61)
Female	15 (42)	7 (50)	8 (44)	7 (39)
BMI				
Median	25.61	25.88	25.61	25.61
BRAF				
Yes	23 (64)	8 (57)	10 (56)	13 (72)
No	12 (33)	6 (43)	7 (39)	5 (27)
No data	1 (3)	0	1 (6)	0
Number of metastatic sites *				
1	5	3	5	0
2	14	8	8	6
3	10	2	4	6
4	7	1	1	6
5	0	0	0	0
Location Visceral metastasis				
Liver	22	6	9	14
Adrenal gland	3	1	2	1
Stomach	2	1	1	1
Colon	2	1	2	0
Small bowel	5	3	4	1
Spleen	2	1	1	1
Pancreas	1	1	1	0
Survival in month				
Median	22.47	28.25	34.1	14.72
95% CI	16.8–28.2	5.0–58.0	17.9–50.5	9.6–19.9
Progress free survival in month				
Median	7.17	9.6	9.6	5.07
95% CI	4.7–9.7	7.4–11.8	8.0–11.2	3.6–6.6

*Initial diagnosis stage 4

### Progression-free survival

Of the 121 patients showing visceral metastases at the time of diagnosis with stage IV MM, 109 (90.1%) patients presented with tumour progress, 8 (6.6%) patients remained stable, and for 4 (3.3%) patients, data was not available. In the group of patients without visceral metastases (229), 175 (76.4%) patients presented with tumour progress, 33 (14.4%) patients remained stable, and for 21 (9.2%) patients, no data was available. Progress appeared in median 6.23 (95% CI: 5.31–7.15) months after systemic first-line therapy if the patient did not present with visceral metastases and after 4.63 (95% CI: 3.81–5.46) months if the patient already showed visceral metastases (*p* = 0.005).

The group of patients with visceral metastases was further evaluated regarding the oligometastatic status. A total of 103 patients did not show an oligometastatic status, while 18 patients presented in an oligometastatic state. Out of the patients with multiple metastases, 93 patients developed progress, 6 patients remained stable, and for 2 patients, data was missing. In the oligometastatic group, 15 patients showed progress, 1 patient remained stable, and for 2 patients, data was not available.

**Fig. 3:**
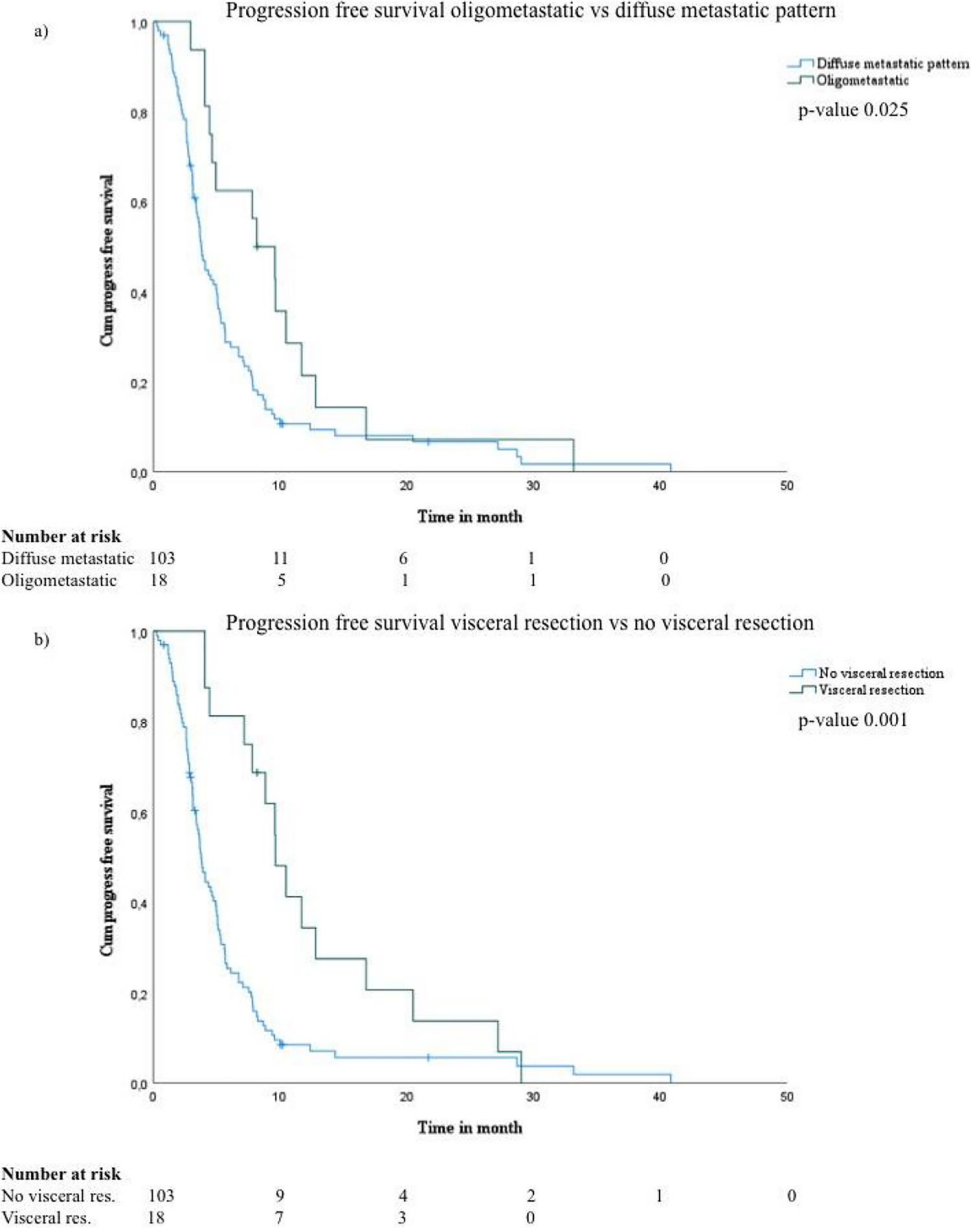
Overview of **a** PFS of patients with stage IV malignant melanoma and visceral metastases with oligometastatic vs. diffuse metastastic pattern and of **b** these patients receiving visceral resection vs. no visceral resection

The PFS was 3.8 months (95% CI: 3.30–4.30) for patients with diffuse visceral metastases vs. 8.17 (95% CI: 4.87–11.46) months for patients in an oligometastatic state (*p* = 0.025) (Fig. 3).

Analysis of patients receiving a visceral resection (18) showed a progress in 15 (83%) patients, no progress in 1 (6%) patient, and for 2 (11%) patients, data was missing.

In this group, 12 patients developed progress after the surgery. Progress appeared in median 7.0 months (95% CI: 1.00–29.00) after the resection.

For this group, the PFS was 9.6 (95% CI: 8.04–11.22) months vs. 3.8 (95% CI: 3.27–4.27) months for the group that did not receive surgical treatment (*p* = 0.001) (Fig. 3).

Matched pair analysis showed a significant impact (*p* = 0.019) on PFS in the surgical group with a hazard ratio (HR) of 0.41, indicating a risk reduction of 59% by the treatment (95% CI: 0.19–0.86) (Table 5). Other analysed variables did not indicate a significant impact on PFS in matched pair analysis.

**Table 5:** Results of matched pair analysis: variables analysed in OS and PFS

OS in PSM				PFS in PSM		
Prognostic variables	*P*-value	Hazard ratio	95% CI	*P*-value	Hazard ratio	95%CI
Oligometastatic	0.025	0.34	0.13–0.87	0.599	0.81	0.37–1.77
Visceral resection	0.016	0.35	0.15–0.84	0.019	0.41	0.20–0.86
BMI	0.668	1.03	0.91–1.16	0.676	1.02	0.93–1.13
BRAF mutation	0.757	1.16	0.45–2.97	0.314	0.67	0.30–1.47
BRAF inhibitor	0.142	1.95	0.80–4.75	0.942	0.97	0.47–2.02
MEK inhibitor	0.603	0.79	0.32–1.92	0.409	0.72	0.33–1.58

## Discussion

In the face of the rising incidence of MM, treatment of oligometastatic melanoma will become one of the significant challenges of oncological medicine and surgery in the future. Considerable progress has already been made in the last 40 years. With an overall median survival estimated at 5.8 months pre-1985 and 8.9 months between 1985 and 2000 including all available treatment options in stage IV MM, this study shows an increase in prognosis for stage IV MM patients [23] with an overall median survival of 14.7 months, even with visceral metastases, for patients treated between 2006 and 2017. Mainly this study was able to show the benefit for patients with oligmetastatic disease stage IV MM that could be treated with a visceral tumour resection. According to current literature, visceral metastases have an inferior prognosis compared to metastases of the lymphnodes or the skin [24]. A further factor to be taken into account is the differentiation between a diffuse pattern and an oligometastatic pattern in MM. Our analysis showed that patients with ≤ 5 metastases seem to have a significantly better prognosis than patients with a diffuse pattern of metastases. This result is not only shown in terms of OS but also with a significant elongation of PFS in patients with oligometastatic disease. Many patients could be passing through a state of oligometastatic disease before being in a diffuse metastatic condition. It is interesting to further investigate if these groups of patients will profit from different treatment regimes. We did not include patients with complete resection of diffuse tumour spread in our cohort, as the highest number of resected metastases was 3. Certainly, a higher number of resections could technically be carried out, but this is not common clinical practice. The survival benefit of the surgical group could be associated with the status of being oligometastatic instead of being in a diffuse state of tumour spread. On the other hand, the favourable prognosis of the oligometastatic disease might be due to the high percentage of patients applicable for surgical resection.

Although stage IV MM is a multiloculary or an oligometastatic disease, the resection of visceral metastases could lead to a considerable gain in median survival time. This was also the case for the PFS, which can be a useful indicator and more reflective of the patients quality of life in certain cases. Continuative studies investigating quality of life after resection of visceral metastases could lead to valuable additive information for clinical decisions.

As shown by previous studies, the prognosis is depending on the exact location of the metastasis [24]. Visceral metastases are mostly associated with diffuse tumour spread and decreased survival rates [25]. While many of the patients in this study showed visceral metastases, 15 (68%) showed an oligometastatic disease pattern, making this cohort unique compared to cohorts previously analysed in literature. The resection of visceral metastases might be a particular powerful tool, if it is used in an early stage of metastasised MM. It could also lose its effectiveness, if it is carried out in advanced stages of MM. This illustrates the importance of early detection of distant metastases and careful follow-up of patients with MM and malignant disease in general.

Consecutively, if the detection of metastases further leads to a resection of the metastasis, it is of utmost importance to carry out a complete resection [26]. After an R1/R2-resection, the prognosis was reduced by more than a third in the surgical group compared to the patients with a R0 resection of the tumour.

Out of the 121 patients, which were diagnosed with visceral metastases in this cohort, only 18 were eligible for surgical resection. A total of 84% of the patients needed different, multimodal treatment options. While stage IV patients commonly profit from systemic therapy, this effect was not visible in the surgical group. Although ¾ of the patients in the surgical group did receive some kind of systemic treatment, there was no difference in survival when comparing systemic treatment prior to or following surgery. Furthermore, patients receiving systemic treatment showed a shorter survival. A reason for that might have been an overall worse general condition, if they were in need of systematic treatment at all. Even though in matched-pair analysis, receiving BRAF- or MEK-inhibitors did not lead to a significant impact on OS. The effects of these treatments in our analysis might be lost due to the small number of patients treated. The results are difficult to discuss, because the rare event of visceral resections in stage IV melanoma made it necessary to gather data from a long period of time to include an acceptable amount of patients. Even prospective data needed 9 years at 18 centers to find only 20 patients that received a visceral resections for melanoma metastatis [1].

Successful treatment of patients with oligometastatic malignant melanoma may include any available treatment option. Visceral resection in oligometastatic melanoma already showed a significant benefit in survival when compared to non-surgical treatment. With improving technical possibilities in the surgical treatment of metastatic lesions, e.g. laparoscopic liver resection and image-guided local ablations, more patients could be eligible for a surgical approach in the future. The options for neoadjuvant and adjuvant systemic treatments are also still expanding in a promising way.

This study has some limitations. The small number of patients included in the surgical group as well as in the oligometastatic group demands a careful discussion of the data. The majority of patients with stage IV MM is not treated at a tertiary center. A visceral resection or local therapy options for visceral metastases might be missed out, although the number of patients that may benefit from this treatment option might be much higher. Another limitation is the small number of patients with visceral resections led to a long time span we used for patient inclusion. The heterogenity in the systemic treatment during that time period may as well influence the outcome. Many of the therapeutics of the current era, such as Nivolumab, were not available at the time the first patients of our cohort were receiving their therapy. This also makes the interpretation of the generalisability difficult to discuss. Still our data suggests that in combination with a targeted immunotherapy, especially in BRAF-positive tumour, visceral resection could provide a promising additional therapeutic option for patients. As seen in different cancer entities already, even in the same patient, different metastases might have different sensitivities to the systemic treatment due to tumour heterogenitiy [27]. The visceral resection of a single or few metastases not responding to systemic therapy due to parallel metastatic evolution including escape mechanisms in an overall sensitive patient with a diffuse pattern of metastatic disease might even further expand the indication for visceral resections.

To further adress this question, randomised controlled trials should be implemented. Based on our data in combination with the available literature, the resection of visceral metastases may have a fundamental position in the treatment of oligometastatic malignant melanoma in the future.

## Conclusion

The resection of visceral metastases is a feasible treatment option in advanced malignant melanoma. Especially, patients with an oligometastatic disease could benefit from surgical treatment.

### Supplementary Information

Below is the link to the electronic supplementary material.

Supplementary file1 (PDF 47 KB)

Supplementary file2 (DOCX 45 KB)
