# Supplementary material for: Oligometastatic disease and visceral resections in advanced malignant melanoma: a propensity-matched analysis
Source: Langenbecks Arch Surg. 2023 Jan 21;408(1):53. doi: 10.1007/s00423-023-02804-9 (PMC9867670; doi:10.1007/s00423-023-02804-9)
Supplement: Supplementary file 1 — Supplementary file1 (PDF 47 KB) [file 423_2023_2804_MOESM1_ESM.pdf]

	Overall survival	Progress free survival
Visceral resection	p=0.222	p=0.171
Oligometastases	p=0.931	p=0.265
BMI	p=0.265	p=0.364
MEK-Inhibitor	p=0.569	p=0.570
BRAF-Mutation	p=0.757	p=0.140
BRAF-Inhibitor	p=0.161	p=0.164

Proportional hazards assumption testing by introducing interaction with log-time as a time-dependent covariate.
